# Data Processing and Machine Learning for Assistive and Rehabilitation Technologies

**DOI:** 10.3390/bioengineering12010070

**Published:** 2025-01-15

**Authors:** Andrea Tigrini, Agnese Sbrollini, Alessandro Mengarelli

**Affiliations:** Department of Information Engineering, Università Politecnica delle Marche, Via Brecce Bianche 12, 60131 Ancona, Italya.mengarelli@staff.univpm.it (A.M.)

This Special Issue (SI), “Data Processing and Machine Learning for Assistive and Rehabilitation Technologies”, aimed to collect cutting-edge research papers that frame how data-driven approaches and machine learning techniques are advancing the field of assistive and rehabilitation technologies. Under this SI, a total of nine contributions were collected [1,2,3,4,5,6,7,8,9]. The selected papers tackle diverse challenges in digital healthcare, ranging from neurodegenerative disease detection, stroke rehabilitation, sleep disorder diagnostics, and robotic therapies. All the contributions published in this SI demonstrated the potential carried by embedding machine learning and data processing technology in assistive and rehabilitation science to create new paradigms of patient treatment, management, and interaction with modern assistive devices.

Despite the high variety of topics, three main research lines are recognizable as of interest to the research community, underlining the need to introduce artificial intelligence (AI) technologies as a tool to solve the increasing challenges that the healthcare system is facing.

The first topic highlighted by the published papers regards the introduction of such tools for the detection of different diseases affecting the central nervous system or the musculoskeletal system [2,3,5,6,8]. Under this framework, the SI demonstrated once more that different sources of data, from brain imaging to posturographic data, can be valuable to create models that are able to predict pathological conditions in incoming patients. Indeed, Salehi and colleagues [3] explored the potential of long short-term memory (LSTM) networks for Alzheimer’s disease identification from magnetic resonance imaging (MRI), whereas in Dindorf et al. [2] an explainable AI model was leveraged for achieving postural deficits diagnosis. In this case, both deep and machine learning systems can be helpful to clinicians by supporting their screening and by looking for hidden patterns that are not considered in the standard clinical scenario. In addition, sleep disorder diagnosis was a specific aspect considered within this SI, by the work of Alattar et al. [8], who thoroughly reviewed the most recent and cutting-edge AI-based technique in this field. The second area regards the field of AI applied in rehabilitation from stroke recovery. In this framework, the published contributions showed how to process data from wearable devices (e.g., electromyographic and inertial measurement unit signals) and how deep learning and hybrid models can be efficient in assessing movement abnormalities and guiding assistive hand therapy in stroke patients [4,9]. In the work by Bonanno et al. [9], the neural plasticity associated with rehabilitative paths based on robotic devices has been reviewed by focusing on those papers where neurofunctional correlates were specifically treated. The last area that received attention from the SI regards the field of applying machine learning models to decode human movement intention for assistive robotics control [1,7,9]. Furthermore, in this context, the potentiality of AI was exploited starting from different sources of data, including near-infrared to neuroimaging and EMG information.

Finally, it is important to underline that the backgrounds of all authors who contributed to the SI are primarily academic. This highlights the role of academicians in steering attention toward the development of theoretical frameworks and the feasibility evaluation of applying the AI models in this field of digital health technology. However, we would stress again the importance of attracting an industry point of view, and this will be a primary aim of the next Special Issue we are launching for MDPI’s *Bioengineering*.
